# Copolymers of bipyridinium and metal (Zn & Ni) porphyrin derivatives; theoretical insights and electrochemical activity towards CO_2_†

**DOI:** 10.1039/d1ra01945g

**Published:** 2021-06-02

**Authors:** Sachin Kochrekar, Ajit Kalekar, Shweta Mehta, Pia Damlin, Mikko Salomäki, Sari Granroth, Niko Meltola, Kavita Joshi, Carita Kvarnström

**Affiliations:** Turku University Graduate School (UTUGS), Doctoral Programme in Physical and Chemical Sciences FI-20014 Turku Finland; Turku University Centre for Materials and Surfaces (MatSurf), Department of Chemistry, University of Turku Vatselankatu 2 FI-20014 Turku Finland carkva@utu.fi; Physical and Materials Chemistry Division, CSIR-National Chemical Laboratory Pune 411008 India; Academy of Scientific and Innovative Research (AcSIR) Anusandhan Bhawan, 2, Rafi Marg New Delhi 110001 India; Laboratory of Materials Science, University of Turku FI-20014 Turku Finland; ArcDia International Oy Ltd Lemminkäisenkatu 32 FI-20521-Turku Finland

## Abstract

This study reports the electropolymerization of novel keto functionalized octaethyl metal porphyrins (Zn^2+^ and Ni^2+^) in the presence of 4,4′-bipyridine (4,4′-bpy) as a bridging nucleophile. The polymer films were characterized by electrochemical, spectroscopic (UV-Vis, XPS, FT-IR and Raman spectroscopy) and imaging (AFM and SEM) techniques. The absorption and electronic spectra confirm the presence of both porphyrin and 4,4′-bipyridine units in the film. The surface morphology reveals homogeneous film deposition with average roughness values of approx. 8 nm. The theoretical studies performed offered insights into the interplay of different metal centres (Zn^2+^ and Ni^2+^) and the keto functionality of the porphyrin unit in the formation of copolymer films. The electrochemical interaction of polymer films with CO_2_ suggests a reversible trap and release of CO_2_ with low energy barriers for both the polymers.

## Abstract 2

Electropolymerization of keto functionalized porphyrins and 4,4′-bipyridine.

## Graphical abstract

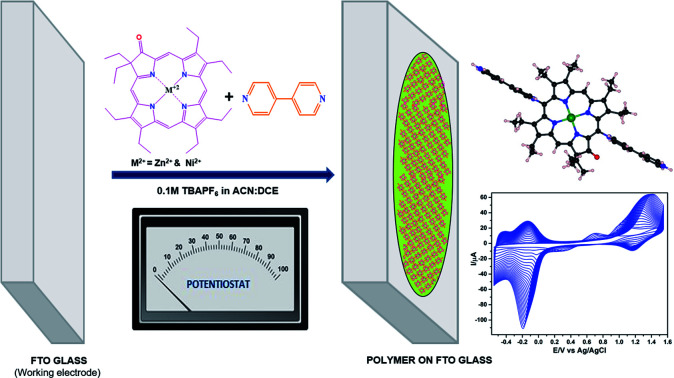

## Introduction

Remarkable development has been seen over the past few decades for compounds containing transition metal complexes due to their versatile conjugated π electron molecular structures and variable catalytic and electronic properties.^1–3^ Catalysts based on metal–nitrogen–carbon (M–N–C) extended structures bear the perceptible advantage of well-defined, efficient and robust molecular structures, allowing functionalization and customization at the atomic level to improve the overall catalytic performance and ease for mechanistic study.^4,5^ Arguably, nature is the best source of inspiration for modern technology. Combining the best features of bioinspired and manmade transition metal complexes those including porphyrins, phthalocyanines, cyclam and their analogues pave a new route towards efficient catalyst for a broad range of reactions.^3,6,7^

Porphyrin a N4 macrocycles are an excellent electronic mediator and are widely studied as they display interesting activity in homogeneous media and confined to electrode surface when used as electro catalyst.^8^ Their properties are also modulated by varying the metal in the ring centre. Metalloporphyrins offer rich and reversible redox properties when confined to electrode surface and tends to improve utilization of active sites and promote electron transport reactions.^9^ In addition to these attributes, the functionalized porphyrin has been recognized to offer drastically altered chemical, molecular and physical properties.^10^ The functionalized porphyrins in the form of metal–organic framework and covalent polymer attracts great attention in material science due to high surface area, an important property in gas storage and capture.^11^ The functional groups are introduced either at the peripheral or the core of the macrocycles, thereby changing aromaticity and degeneracy of the electronic structure.^12^

To date, methods such as covalent self-assembly, spin coating, drop-casting, electropolymerization, electrospray and electrostatic self-assembly are employed to immobilize different porphyrins on electrode surfaces.^13,14^ Among them, electropolymerization allows the formation of polymer films with enhanced stability and high surface area; moreover, compact layers with control over thickness can be produced.^15,16^ Different strategies have been demonstrated to electropolymerize porphyrin films with high density on the electrode surface.^10,17–20^ Ruhlmann and coworkers reported an easy and simple method to electropolymerize porphyrins without electroactive substituents in presence of Lewis bases with at least two active sites as bridging nucleophiles.^21,22^ The key step was attributed to the nucleophilic reaction between the Lewis bases and the generated dication of the octaethylporphyrins. Also, polymer properties like conductance through the extended conjugation can be varied by changing bridging nucleophile or functionalizing the porphyrins, keeping at least two accessible *meso* positions.^10,23–25^

Metalloporphyrins immobilized electrodes are extensively studied for their profound usage in diverse application. In this work, we demonstrate the electropolymerization of β-keto functionalized metal porphyrins (NiOEPK and ZnOEPK) in the presence of a bridging ligand 4,4′-bpy (4,4′-bipyridine) on the FTO surface. In the search of earth abundant and non-precious metal based system the studies of the two particular porphyrin units *i.e.* ZnOEPK and NiOEPK are interesting as Ni porphyrins are ring oxidized along with the central metal, unlike Zn porphyrin with two ring oxidation steps with unreacted central metal.^21^ Bipyridine complexes are also mostly studied molecular catalysts. 4,4′-bpy functioning as bridging nucleophile and also as a peripheral substituent is crucial, as it has overall influence on the electronic structure of the system, which it turn dictates its electrochemical activity.^26–28^ UV-Vis, FTIR, Raman and X-ray photoelectron spectroscopy along with AFM, SEM and EDX establishes the grafting of 4,4′-bpy with the porphyrin to form polymer films on the electrode surface. DFT calculations were performed to investigate the electropolymerization process of β-keto functionalized porphyrin (ZnOEPK and NiOEPK) with 4,4′-bpy and understand the effect of the central metal atom and keto functionalization on polymer formation. We also looked into the electrochemical interaction of deposited polymer films with N_2_ and CO_2_. Metalloprophyrins are known to possess favorable properties regarding CO_2_ electrocatalysis. However, with this type of polymer there are also other not so well-known electrochemical interactions with CO_2_. Addition of CO_2_ proceeds *via* carbamate formation involving the 4,4′-bpy substituents. This process proceeds with reversible trap and release of CO_2_ with low energy barriers.

## Experimental section

### Materials

All chemical reagents and solvents used were analytical grades or higher. Zinc octaethyl porphyrin ketone (ZnOEPK) and nickel octaethyl porphyrin ketone (NiOEPK) were synthesized according to the previous report.^12,29^ 4,4′-Bipyridine (4,4′-bpy) [Alfa Aesar 98%], ferrocene [Aldrich 99%], tetrabutylammonium hexafluorophosphate (TBAPF_6_) [TCI 98%] were used without further purification unless stated. TBAPF_6_ was dried in a vacuum oven at 70 °C for 2 h before use. Anhydrous acetonitrile (ACN) [Sigma Aldrich 99.9%], dichloromethane (DCM) [VWR chemicals 99.8%] and dichloroethane (DCE) [Alfa Aesar 99%] was stored over 4 Å molecular sieves in an argon-filled glove box. Nitrogen (N_2_), carbon dioxide (CO_2_) and argon (Ar) were acquired from AGA-Finland.

### Electrochemical measurements

Cyclic voltammetry was performed using a conventional three electrodes system and an Autolab PGSTAT101 potentiostat. One-sided fluorinated indium tin oxide (FTO) [Pilkington] with a sheet resistance of 8.1 Ω sq^−1^, was used as a working electrode. Before use, it was cleaned by ultrasonication in acetone, ethanol and water for 10 min followed by plasma cleaning for 3 min. A coiled platinum wire was used as a counter electrode. All potentials are reported *vs.* a silver chloride coated silver wire (Ag/AgCl) pseudo reference electrode prepared galvanostatically in saturated KCl(aq) and calibrated against ferrocene/ferrocenium couple, *E*_ref_ = 0.45 V in 0.1 M TBAPF_6_ in acetonitrile.

### Electropolymerization

All the polymer films were formed by 25 iterative potentiodynamic cycles in 5 × 10^−4^ M porphyrin (NiOEPK and ZnOEPK) and 5 × 10^−4^ M 4,4′-bpy solution in 0.1 M TBAPF_6_ in (4 : 1) DCE : ACN. The potential range was in between −0.6 to 1.6 V *vs.* Ag/AgCl at a scan rate of 200 mV s^−1^. Electropolymerization of porphyrin in the presence of an appropriate bridging nucleophile leads to the growth of a polymer film.^21,22^ The obtained polymer films were rinsed with ACN followed by quartz-distilled water to remove traces of unreacted starting material on the electrode surfaces and preserved in a desiccator for further use.

### Interaction with CO_2_

To understand the interaction of carbon dioxide with the keto-functionalized porphyrin polymer films cyclic voltammetry (CV) measurements were performed in three-electrode customized airtight single compartment cell. The polymer films of ZnOEPK and NiOEPK on/or blank FTO was used as a working electrode, Pt wire as a counter electrode and Ag/AgCl as a quasi-reference electrode. The studies were performed in deaerated and dry 0.1 M TBAPF_6_ in ACN solution pre-saturated with either N_2_, CO_2_ or Ar for 30 min at a scan rate of 100 mV s^−1^ in the potential range of 0 to −1.35 V *vs.* Ag/AgCl.

### Spectroscopic measurements

UV-Vis absorption spectra of the monomer dissolved in DCM and polymer film on the electrode surface were recorded by an Agilent Cary 60 UV-Vis spectrophotometer with blank FTO as a reference for background correction.

FTIR spectra of the polymer films were measured by a Bruker Vertex 70 FTIR spectrometer with MCT detector cooled with liquid nitrogen. Each spectrum was recorded at 55° incidence angle relative to a surface normal using a Harrick Seagull accessory, compiling 248 scans at 4 cm^−1^ spectral resolution.

Raman spectra were obtained by Renishaw inVia confocal Raman microscope equipped with a CCD detector and a Leica microscope. Spectra were recorded with 50× objective, 1200 l mm^−1^ grating and using a 532 nm diode laser as the excitation source with 10 mW laser power and accumulation of 1.

XPS measurements of porphyrin film on the FTO substrates were recorded by PerkinElmer PHI 5400 spectrometer using Mg Kα radiation (1253.6 eV). All the binding energies were calibrated to the peak position of the main C 1s signal at 284.6 eV of the porphyrins. All the XPS spectra were fitted using and Voigt line shape (convolution of Gaussian and Lorentzian) after subtraction of Shirley background fitting was performed using the Igor Pro SPANCF curve fitting macro package. Broad energy range survey scans were performed to determine the atomic concentration and to check possible impurities with a pass energy of 89.45 eV. Higher-resolution spectra were also collected for selected core-levels. For those, pass energy of 37.75 eV was used.

### Morphology of the films

AFM studies were conducted by Veeco diCaliber on the modified electrode surface using silicon tipped cantilever operating in the tapping mode. The image processing and analysis have been carried out using the WSxM 4.0 software. Surface morphology was evaluated by Thermo Scientific Apreo S (FE-SEM) with Oxford Instrument Ultim Max 100 EDS spectrometer.

### Theoretical studies

To understand the polymerization of keto functionalized octaethyl porphyrin with 4,4′-bpy, the most favourable position for the nucleophilic interaction of first 4,4′-bpy was determined by optimizing all four different *meso* positions. The most stable structure among these four structures was then optimized for the second 4,4′-bpy to understand the variation in polymerization due to change in the central atom. All the calculations were carried out within the Kohn–Sham formulation of DFT. Projector augmented wave potential^30,31^ was used, with Perdew–Burke–Ernzerhof (PBE)^32^ approximation for the exchange–correlation and generalized gradient approximation,^33^ as implemented in-plane wave pseudopotential based code, VASP.^34–36^ Cubic simulation cell, with images separated by at least 15 Å of vacuum in all three directions, was used. The porphyrin structure was created using Quantumwise-VNL-2017.1.^37^ For each SCF cycle, energy convergence criteria of 10^−4^ eV were implemented. For structural optimization, force cutoff of 0.01 eV Å^−1^ was used. The M–N distances of the crystallographically characterized ZnOEP and NiOEP is 2.05 Å and 1.96 Å respectively which matches exactly with the previously reported results.^38,39^ HOMO and LUMO of the systems were plotted with isosurface value of one-tenth of its maxima.

## Results & discussion

### Electropolymerization

**Fig. 1:**
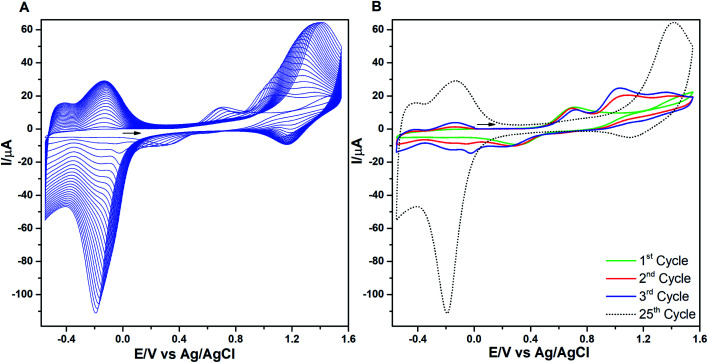
(A) Cyclic voltammograms recorded during electropolymerization of NiOEPK (2.5 × 10^−4^ M) containing 4,4′-bpy (2.5 × 10^−4^ M) in 0.1 M TBAPF_6_ in (4 : 1) DCE : ACN in the potential range −0.55 to 1.55 V (25 cycles) using 200 mV s^−1^ as scan rate. In (B) the 1^st^, 2^nd^, 3^rd^ and 25^th^ cycle are shown.

**Fig. 2:**
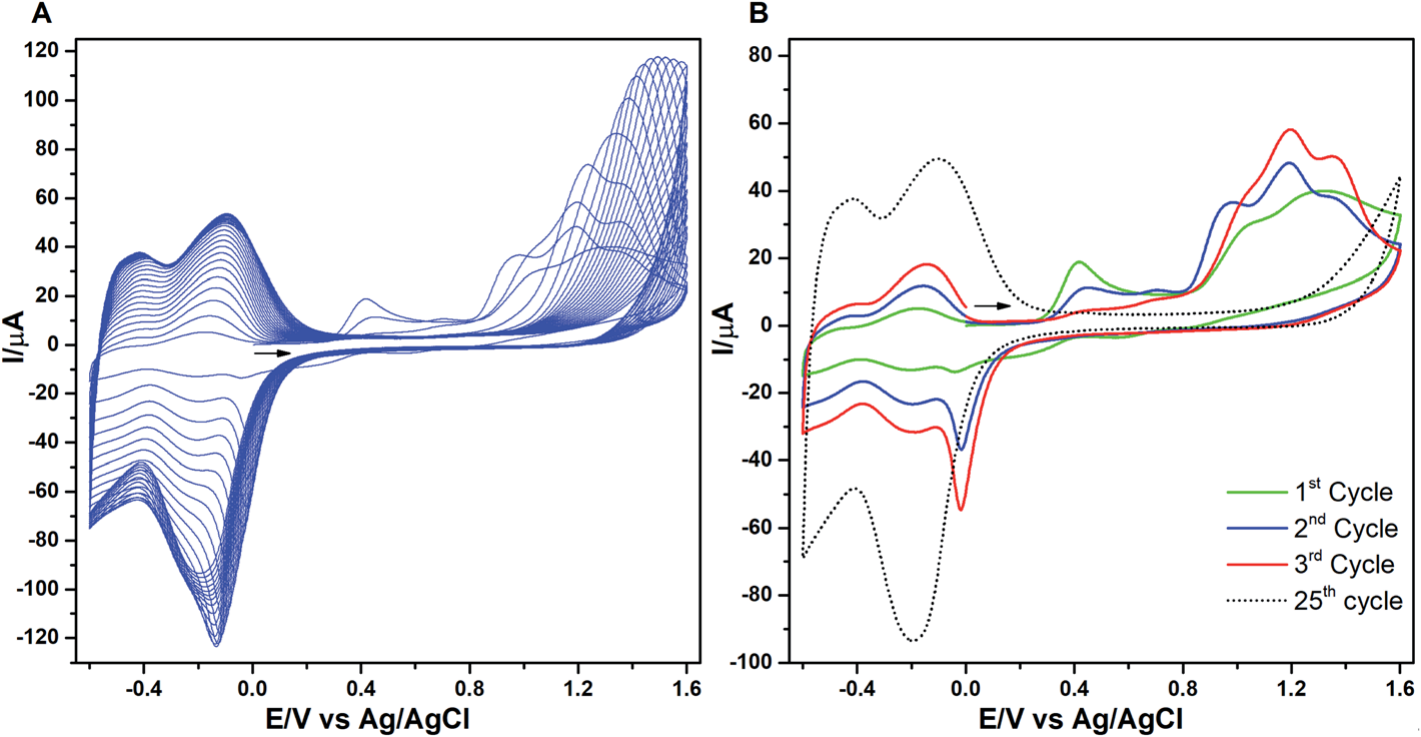
(A) Cyclic voltammograms recorded during electropolymerization of ZnOEPK (2.5 × 10^−4^ M) containing 4,4′-bpy (2.5 × 10^−4^ M) in 0.1 M TBAPF_6_ in (4 : 1) DCE : ACN in the potential range −0.6 to 1.6 V (25 cycles) using 200 mV s^−1^ as scan rate. In (B) the 1^st^, 2^nd^, 3^rd^ and 25^th^ cycle are shown.

The consecutive voltammetric scans recorded during electropolymerization of metal octaethyl porphyrin ketones (M = Zn^2+^ [ZnOEPK −0.6 to 1.6 V *vs.* Ag/AgCl] and Ni^2+^ [NiOEPK −0.55 to 1.55 V *vs.* Ag/AgCl]) in the presence 4,4′-bpy acting as bridging nucleophile at a scan rate of 200 mV s^−1^ starting from 0 V going in the anodic direction are presented in Fig. 1 and 2. The potential window is optimized for obtaining homogeneous reproducible production of stable polymer films.

**Scheme 1:**
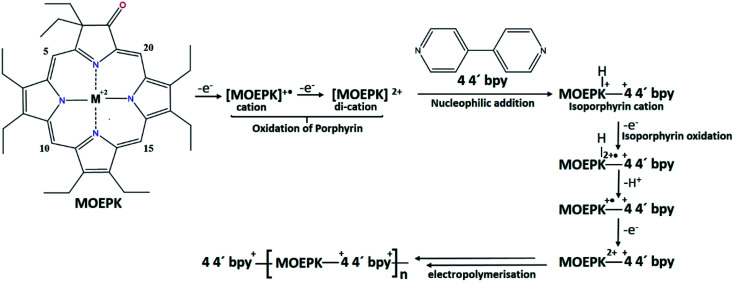
Mechanism for electropolymerization of MOEPK in presence of 4,4′-bpy in 0.1 M TBAPF_6_ in (4 : 1) DCE : ACN.^22,41,44^

During the first cycle, NiOEPK showed one oxidation peak at 0.71 V and an increasing current starting at 1.2 V and on the reverse scan one reduction peak at 0.31 V. From the second cycle on three oxidation peaks can be seen at 0.69, 1.05 and 1.38 V. The first two peaks originate from the cation and dication formation of the metal porphyrin.^22,40,41^ The porphyrin dication being strong electrophile undergoes nucleophilic attack by 4,4′-bpy to form isoporphyrin cation^42^ as depicted in Scheme 1. The third peak is attributed to the reversible oxidation of an isoporphyrin. All these peaks shift towards more positive potential due to the progress of electrode coating, these peaks merge later into one broad peak at 1.41 V. On the reverse scan, the reductive peaks at 0.31, −0.05 and −0.18 V are seen together with an increase in current close to the switching potential, which originates from the second reduction of the 4,4′-bpy. The two later ones are from the reduction of isoporphyrin and bipyridinium respectively and are merging into a major cathodic peak −0.18 V. The peak developed at 0.31 V is the reduction of the oxidized porphyrin in the solution. In ZnOEPK (Fig. 2), three successive one electron oxidation peaks were observed at 0.42, 1.02, and 1.3 V. The first two peaks originate from the cation and dication formation of the porphyrin and the third peak is attributed to the reversible oxidation of an isoporphyrin.^22^ On further iterative scans, the first peak diminishes after a few cycles and the latter two peaks merge into one broad peak that is shifted to a higher potential. In the cathodic direction, the reduction peaks at −0.04 and −0.22 V for ZnOEPK are associated with the reduction of isoporphyrin and bipyridinium, respectively. Upon continuous scans, these peaks merge into a major cathodic peak similar to NiEOPK. In both cases, there is an increasing current starting from −0.4 V which is assigned to the beginning of the second one electron reduction of bipyridium unit. The progressive increase in the peak current with every cycle clearly shows the continues growth and deposition of the polymer film on the electrode surface.^23,43^

### Theoretical studies

**Fig. 3:**
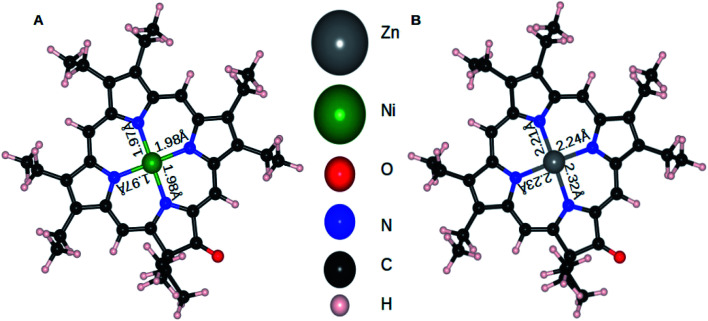
NiOEPK (A) and ZnOEPK (B). Note that Zn gets asymmetrically attached to N atoms whereas very little variation is observed in Ni–N bond-lengths in NiOEPK.

The mechanism for electropolymerization of akin porphyrin species is well established. It occurs *via* nucleophilic attack of the bridging nucleophile at the active *meso* position leading to the formation of polymers. To understand the effect of keto functionalization and metal centres (M = Zn and Ni) on the obtained polymer geometry, DFT studies were performed. First, we will compare the influence of central atom Zn and Ni on the resulting ZnOEPK and NiOEPK structures respectively. The effect of completely and partially filled valance orbitals (as in case of Zn and Ni) reflects in the respective porphyrins structures. Despite Zn having smaller ionic radii than Ni, Zn–N bond length in ZnOEPK ranges from 2.21 to 2.32 Å. Whereas, the bond length of Ni–N in NiOEPK showed hardly any variation (1.97 Å to 1.98 Å). Also, it accounts for the blue shift in the spectra. Further, all M–N bond lengths are almost equal in NiOEPK, unlike that of ZnOEPK (Fig. 3).

**Fig. 4:**
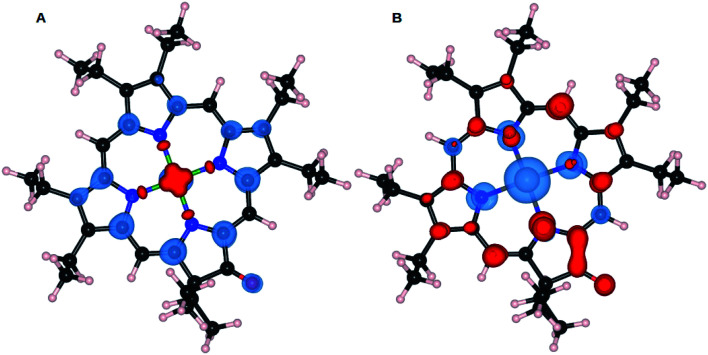
Isosurface of HOMO and LUMO of NiOEPK (A) and ZnOEPK (B). The HOMO are depicted by blue and LUMO by red colour. Variation in spatial distribution of HOMO and LUMO is observed in both the cases is due to variation in the central metal atom.

The significant contribution of the central atom in NiOEPK and ZnOEPK is witnessed from the spatial distribution of HOMO–LUMO in both the systems (Fig. 4). The differences in the HOMO and LUMO explain the variation observed in the preferred *meso* position for the nucleophilic addition of the first 4,4′-bpy nucleophile. The HOMO in ZnOEPK is mostly around the central Zn atom and nearby N and C atoms, whereas in NiOEPK it is more delocalized and spread over most of the porphyrin but categorically excluding N atoms. However, LUMO in ZnOEPK is comparatively more delocalized and prominent around the keto group, while LUMO in NiOEPK is mostly localized near the central metal atom (Fig. 4). DFT studies validate that the first 4,4′-bpy prefers *meso* position near to the keto group for ZnOEPK. Whereas in NiOEPK, energetically most favourable *meso* position is farthest from the keto group (Fig. S5†). This interesting consequences due to the influence of different metal species is understood based on the subtle differences observed in the spatial distribution of HOMO–LUMO of NiOEPK and ZnOEPK (Fig. 4).

**Fig. 5:**
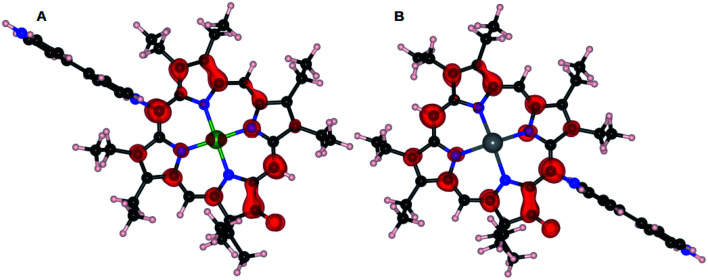
Isosurface of LUMO of NiOEPK–4,4′-bpy (A) and ZnOEPK–4,4′-bpy (B).

The LUMO of NiOEPK and ZnOEPK with the first 4,4′-bpy nucleophile were further analyzed to investigate the nucleophilic addition of the second 4,4′-bpy nucleophile. As shown in Fig. 5, LUMO in both NiOEPK and ZnOEPK are identical. It is interesting to note that LUMO are distributed over the *meso* position opposite to the first 4,4′-bpy which facilitates linear polymerization in both the porphyrins (structure of linear polymerization is shown in Fig. S6†). It should be noticed, that second 4,4′-bpy prefers the opposite position concerning first bpy nucleophile attached to the porphyrins. It turns out to be the farthest *meso* position from keto group in ZnOEPK and the nearest in case of NiOEPK. Further, both 4,4′-bpy units prefer parallel orientation to each other and perpendicular to the plane of the porphyrin unit while proceeding linearly.

### Spectroscopic analysis

**Fig. 6:**
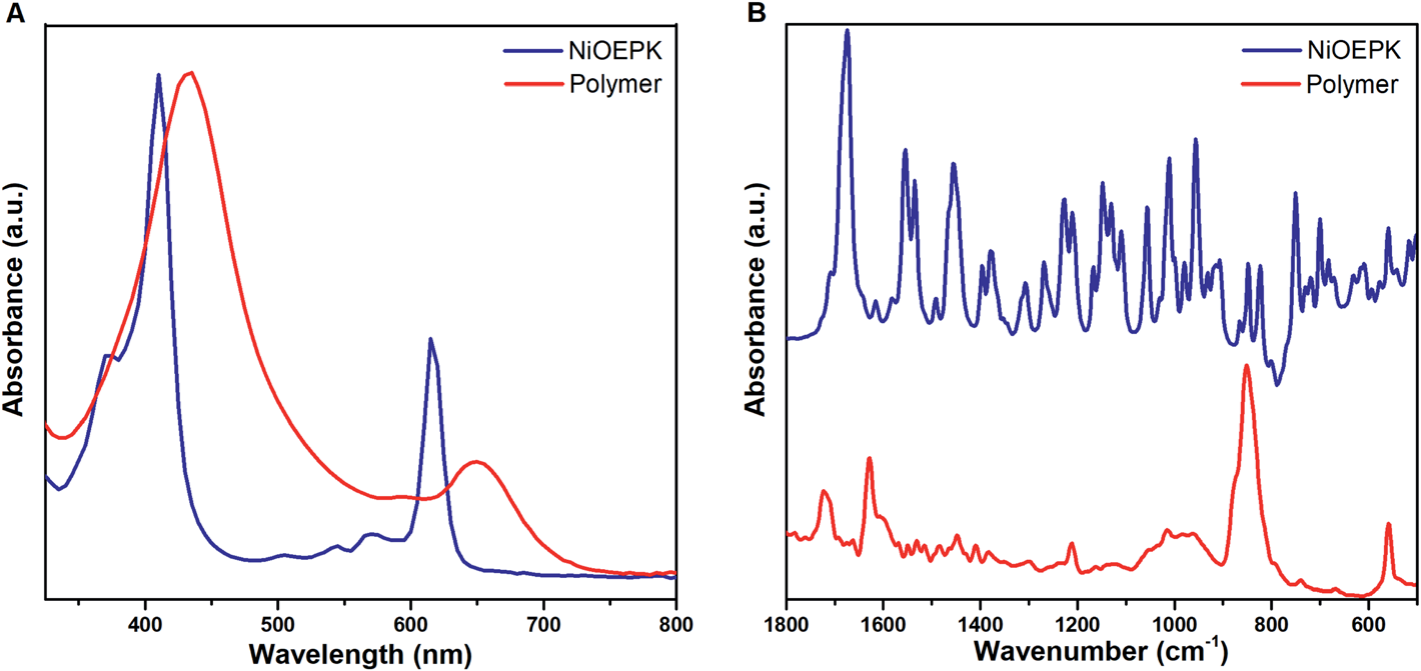
UV-Vis absorption (A) and FT-IR (B) spectra of NiOEPK monomer and polymer film.

The UV-Vis spectra in Fig. 6A and S1A† of the polymer films on FTO and their monomers dissolved in DCM exhibit typical porphyrin traits with a strong Soret band and weaker Q band/s arising due to transition between HOMOs and LUMOs in the metal centre and the substituents.^45–47^ The number of Q bands in the monomer with the introduction of a keto group on the porphyrin backbone is attributed to a decrease in the symmetry of the molecule. The blue shift observed in Ni complex as compared to Zn is due to decreased electron density arising from π bond delocalization with increase energy available for electron transition.^48^ However, the absorption spectra of the polymer films exhibit broader Soret band and one detectable Q band with reduced intensity and red-shifted (Table 1). This effect due to polymerization is well documented by extension in conjugation with the increased number of electron withdrawing pyridinium spacers.^49^ This reflects the change in symmetry with grafting of 4,4′-bpy on the porphyrin along with the integrity of the porphyrin unit in the polymer films.^50,51^

**Table 1:** Comparing UV-Vis spectra of NiOEPK and ZnOEPK in DCM and as polymer film on FTO

Compounds	Solution		Polymer film	
	Soret band (nm)	Q band (nm)	Soret band (nm)	Q band (nm)
	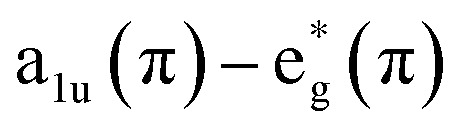	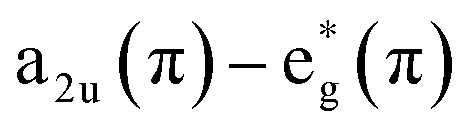	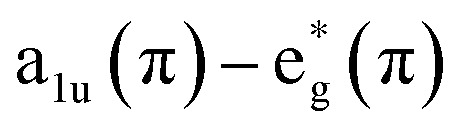	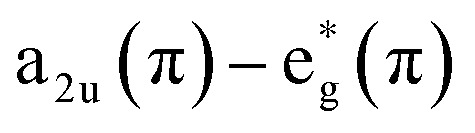
ZnOEPK	415	520, 570 & 620	440	655
NiOEPK	410	505, 545, 570 & 615	435	650

FTIR spectra of NiOEPK monomer and polymer (Fig. 6B) and ZnOEPK monomer and polymer (Fig. S1B†) reveals characteristic modes of vibration from the porphyrin framework. However, the spectrum of the polymer film is much broader with reduced intensity. The intense band at 1722 cm^−1^ is assigned to C=O stretching on the 5 members strained ring. The downshift is attributed to elongated conjugation. The characteristic C=N stretching vibration from the pyridinium ring at 1627 cm^−1^ manifests the presence of 4,4′-bpy in the polymer skeleton.^52^ The weak peaks around 1015, 1213 and 1448 cm^−1^ correspond to C–H rocking vibration, C–N vibration and skeletal vibration from the pyrrole ring, respectively.^52,53^ The peak at 852 cm^−1^ is associated with *meso* C–H out of plane bending vibration.^54^

**Fig. 7:**
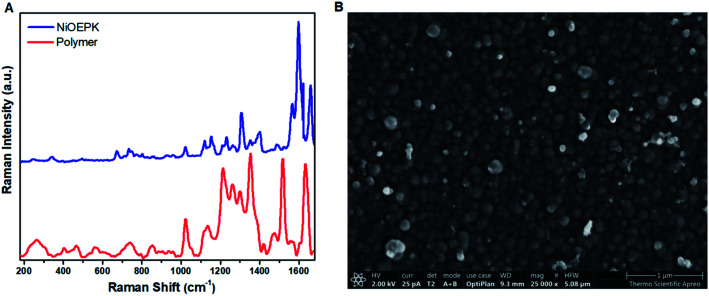
Raman spectra (A) of poly NiEOPK on FTO (red) and NiOEPK monomer (blue) and SEM image (B) of poly NiOEPK.

The formation of a polymer film with porphyrin character on the electrode surface was further shown by Raman spectroscopy. Fig. 7A and S2† demonstrating some characteristic bands around 1400–1670 cm^−1^ range, mainly from the porphyrin skeleton *i.e.* C=C and C=N stretching modes.^46^ For the monomer samples, the influence of M–N bond strength on the C=C and C=N stretching vibrations are evident.^55^ A slight shift in these stretching modes due to polymerization can be observed. In the case of the polymers, there are considerable coupling with stretching vibration arising from the 4,4′-bpy linker. The strong intensity around 1300 cm^−1^ in the polymer films indicates incorporation of 4,4′-bpy in the porphyrin network. The retention of the optical and electronic properties of the monomer unit in the polymer film suggests the Π-conjugated system remain intact on the electrode surface.

**Fig. 8:**
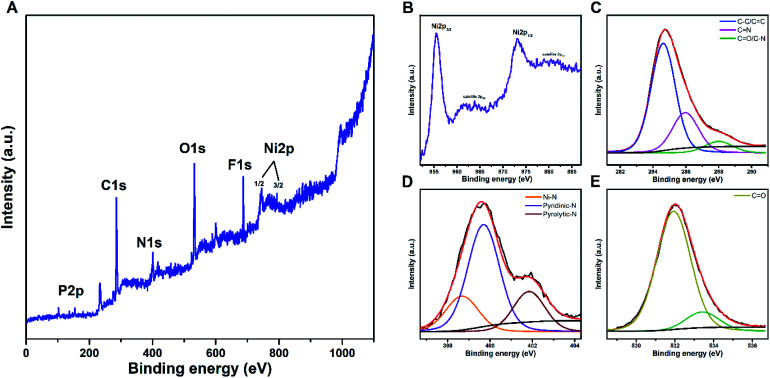
XPS spectra of poly NiOEPK (A), Ni (B), C 1s (C), N 1s (D) and O 1s (E).

The elemental composition and chemical state of the polymeric films were further examined by XPS analysis. The deconvoluted XPS spectra into component peaks are shown in Fig. 8 and S3.† The C 1s XPS spectra are unfolded into three components. The peaks at ∼284.6 and ∼285.9 eV are attributed to the carbon backbone (C–C/C=C and C=N) of the porphyrin films and the one at ∼288.2 eV is characteristic for the C–N and C=O moiety residing on the porphyrin.^47,56^ The photoelectron N 1s spectra also exhibit three distinct signals. Mainly the peak arising at ∼398.6 eV (lower binding energy [BE]) is characteristic of the four nitrogens in the metalloporphyrin (Zn^2+^ and Ni^2+^) framework.^47,57^ The peaks at ∼399.9 and ∼401.9 eV are approximated to the spacer used for interconnecting the porphyrin and to the electropositive pyridine (bpy^+^) nitrogen atom, respectively.^58^ The O 1s spectra manifest the most intense signal to oxygen bound carbon (O=C) on the porphyrin at ∼531.4 eV.^56^ An additional peak recorded at 533.4 eV is due to absorbed oxygen impurities on the film surface.^59^ The XPS spectrum also shows the presence of F 1s and P 2p attributed to the counter ion PF_6_^−^.^59^ These metalloporphyrin films exhibit characteristic BE of the 2p core levels of M(ii) species. In poly ZnOEPK the presence of Zn(ii) centred porphyrin is confirmed by the BE of Zn 2P_3/2_ and Zn 2P_1/2_ at 1021.9 and 1045.1 eV, respectively.^60^ In the case of poly NiOEPK, the presence of Ni(ii) is evidenced by the appearance of 2P_3/2_ and 2P_1/2_ core levels at 854.4 and 873.2 eV, respectively.^45^ The spectrum also incorporates two structureless and very weak shakeup satellites at 862.6 and 881.8 eV ubiquitous in Ni(ii) porphyrin due to relaxation effect.^45,47,57^ The weakness of these signals is in agreement with the diamagnetic nature of Ni(ii) in poly NiOEPK.^45,57^ The similarity in the nature of poly NiOEPK and poly ZnOEPK, except for the central metal species, clearly reflects from the N 1s, C 1s and O 1s spectra.

### Morphology

**Fig. 9:**
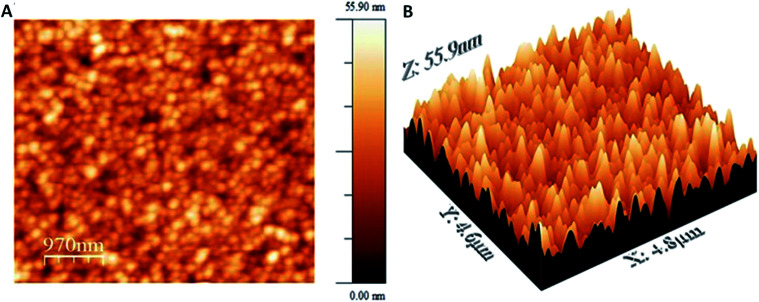
AFM image of poly NiOEPK on FTO electrode as 2D (A) and 3D (B) view.

The AFM and SEM images of the polymer films after 25 cycles (Fig. 7B, 9 and S4†) demonstrates a tightly packed distorted sphere containing rough surface, depicting heterogeneous polymerization of keto functionalized porphyrin across the FTO electrode surface. From AFM the roughness estimated by the root mean squared (RMS) was around 8 nm for the polymer films. Also, the EDX mapping revealed uniform distribution of Ni, N, C and O in the polymer films.

### Interaction between CO_2_ and polymer film

**Fig. 10:**
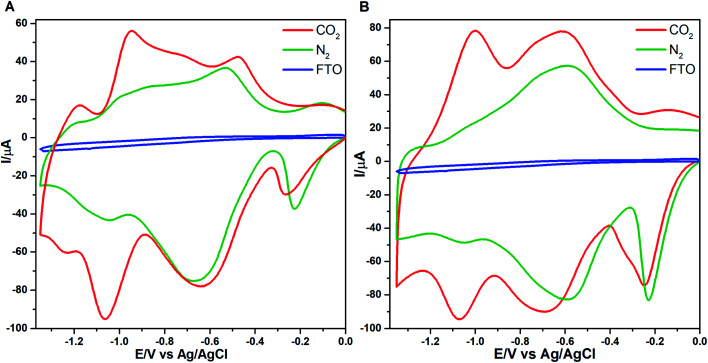
Cyclic voltammograms of poly-NiOEPK (A) and poly-ZnOPEK (B) on FTO surface saturated with CO_2_ and with N_2_ and for 30 min at 100 mV s^−1^ in 0.1 M TBAPF_6_ in ACN. CV of CO_2_ saturated on FTO (blue) for comparison.

To understand the potential as a catalyst, electrochemical interaction between the CO_2_ and the polymer were investigated in the absence (N_2_) and the presence of CO_2_. The measurement including nitrogen-saturated solution showed two distinct reduction peaks (Fig. 10). The first, located at near −0.2 V is attributed to the formation of radical cation of the bpy linker and the second reduction peak observed at approximately −0.6 V is attributed to the second reduction of the bpy linker. The shape and position of the peaks are well in line with similar polymers described in the literature.^22,23,61^ However, there is a minor additional reduction process observable at −1.05 V which is more clearly seen in the case of ZnOEPK. After saturation of the electrolyte with CO_2_, the reduction processes of the bpy linker take place roughly at the same potentials as earlier. Some peak voltage variation is observed because of the possible activity of CO_2_ on the redox active species in the film. However, the most noticeable feature induced by CO_2_ is the apparent activation of the −1.05 V reduction process. The presence of CO_2_ induces one additional reversible redox process in the film. The reversibility and overall shape of the reduction peak, however, does not support the idea of electrocatalytic reduction of CO_2_ by the metal centre of the porphyrin. Further investigation of reversible processes in the negative voltage range is difficult because the film becomes increasingly labile at voltages more negative than −1.3 V. When proceeding beyond this point the reverse oxidation scan does not produce reliable information anymore.

**Scheme 2:**
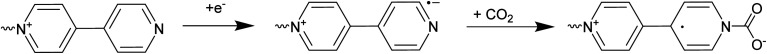
Formation of carbamate intermediate.^62^

In Fig. 10 the CO_2_ induced current growth can be observed in the peak at −1.05 V. It is found that the addition of CO_2_ in bipyridine containing material increases the reduction current of bipyridine^62^ and some bipyridyl derivatives.^63,64^ The Lewis basicity of the unsubstituted pyridyl or bipyridyl nitrogen has shown to support the formation of stable carbamates after reduction to radical form in presence of CO_2_ (Scheme 2).^62,63^

CO_2_ interaction with the polymer film especially the activation of −1.05 V process could be attributed to intermediate carbamate formation in the monosubstituted bipyridines (Scheme 2). The exact structure of the polymer is under discussion but there are possibilities of having bipyridinium functionalities at the end of the chain as well as a side chain. The potential of the carbamate formation has been observed to shift with the bipyridine substitution.^62–64^ Unsubstituted bipyridine has the highest negative formation potential for carbamate formation^62^ while the propyl-substituted bipyridine has formation potential close to the values observed here.^63^

The presence of electroactive bipyridyl substituents was previously detected by Schaming *et al.* form an additional reduction wave observed at 0.3 V more negative than first bipyridine spacer reduction.^22^ In our data, however, the same cannot be clearly detected. That might be due to different structure *i.e.* the keto functionalization of the porphyrin monomer. There are also previous papers concerning the redox properties of pyridinium and bipyridinium substituents. A porphyrin monomer substituted with four pyridiniums^65^ or bipyridiniums^66^ leads into four separate reduction waves at a considerably high negative voltage, which is an indication of interaction between the substituents. However, in a porphyrin pentamer having only viologen linkers and no side groups, all the first and second reductions of the linkers take place at the same time at considerably lower voltages,^66^ which is an indication of independent nature of the linkers.

## Conclusion

We have demonstrated a controlled and reproducible electrosynthesis of a novel keto functionalized porphyrin and 4,4′-bpy copolymer. The spectroscopic data of the polymer films resemble those of the monomer unit except for slight shifts and broadening of bands in the polymer film, indicating that porphyrin molecules are preserved and remain intact in the film. The polymer films exhibit similar topographical characteristics. Theoretical studies provide insight into the effect of the central metal atom and the keto functional group on the mode of polymerization. The nucleophilic addition of 4,4′-bpy in ZnOEPK occurs at 20^th^*meso* position close to the keto group whereas in NiOEPK it is attaching at 10^th^*meso* position opposite to the keto group. However, with the introduction of a second 4,4′-bpy unit coupling occurs perpendicular to the plane of porphyrin, opposite to the first unit leading to polymers with linear orientation considering theoretical studies were performed for the initial two 4,4′-bpy units.

The polymer films were investigated by cyclic voltammetry to understand the electrochemical interaction with CO_2_ in ACN. The film response to CO_2_ in comparison to N_2_ infers the immediate interaction of CO_2_. The interaction is seen as an activation process at −1.05 V and could be associated with the formation of intermediate carbamate. The interaction can also be utilized as an activation route towards CO_2_ reduction or as controlled electrochemical trapping and release of CO_2_.

## Conflicts of interest

There are no conflicts to declare.

## Supplementary Material

RA-011-D1RA01945G-s001
