# Supplementary material for: Copolymers of bipyridinium and metal (Zn & Ni) porphyrin derivatives; theoretical insights and electrochemical activity towards CO2
Source: RSC Adv. 2021 Jun 2;11(32):19844–55. doi: 10.1039/d1ra01945g (PMC9033822; doi:10.1039/d1ra01945g)
Supplement: RA-011-D1RA01945G-s001 [file RA-011-D1RA01945G-s001.pdf]

Electronic Supplementary Material (ESI) for RSC Advances.
This journal is © The Royal Society of Chemistry 2021

Supporting information

Copolymers of bipyridinium and Metal (Zn & Ni) porphyrin derivatives, theoretical insight and their electrochemical activity towards CO_2

Sachin Kochrekar,[a,b] Ajit Kalekar,[b] Shweta Mehta,[c,d] Pia Damlin,[b] Mikko Salomäki,[b] Sari Granroth,[e] Niko Meltola,[f] Kavita Joshi,[c,d] Carita Kvarnström[b,*]

[a]Turku University Graduate School (UTUGS) Doctoral Programme in Physical and Chemical Sciences, FI-20014 Turku,Finland

[b]Turku University Centre for Materials and Surfaces (MatSurf), Department of Chemistry, University of Turku, Vatselankatu 2, FI-20014 Turku, Finland.

[c]Physical and Materials Chemistry Division, CSIR-National Chemical Laboratory, Pune 411008 India.

[d]Academy of Scientific and Innovative Research (AcSIR), Anusandhan Bhawan, 2, Rafi Marg, New Delhi – 110001, India.

[e]Laboratory of Materials Science, University of Turku, FI-20014, Turku, Finland.

[f]ArcDia International Oy Ltd, Lemminkäisenkatu 32, FI-20521-Turku, Finland.

Corresponds to: carkva@utu.fi

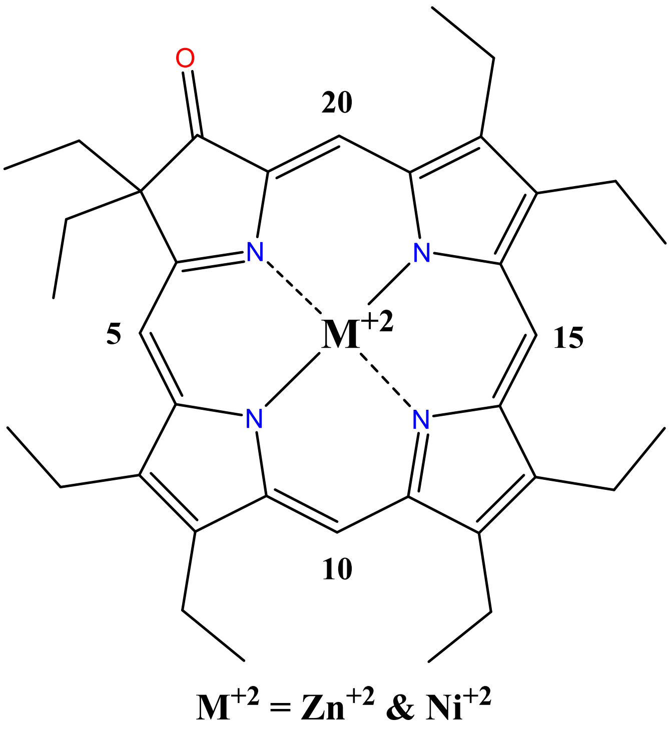

Scheme S1: representation of metal (Zinc and Nickel) Octaethyl porphyrin ketone.

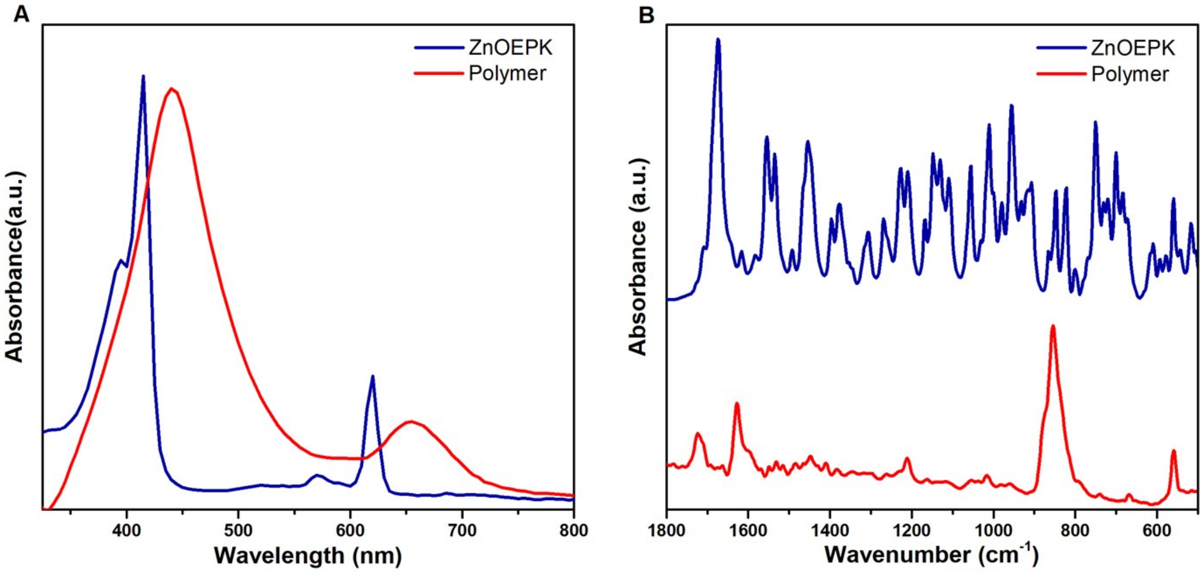

Fig S1 A) UV-Vis absorption(A) and FTIR(B) spectra of ZnOEPK monomer and copolymer film .

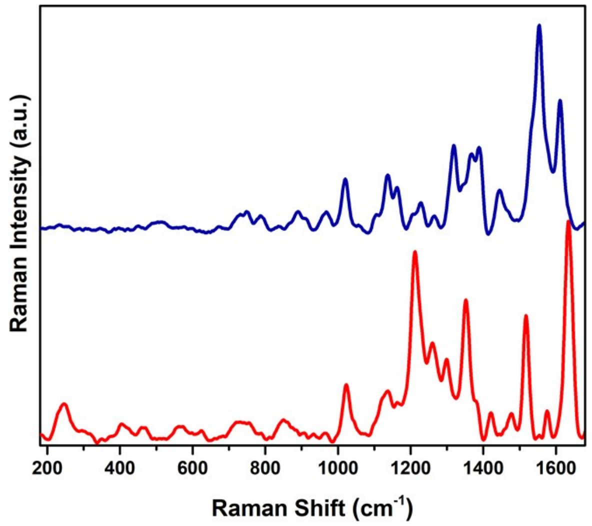

Fig S2 Raman spectra of poly ZnEOPK on FTO (red) and ZnOEPK monomer (blue)

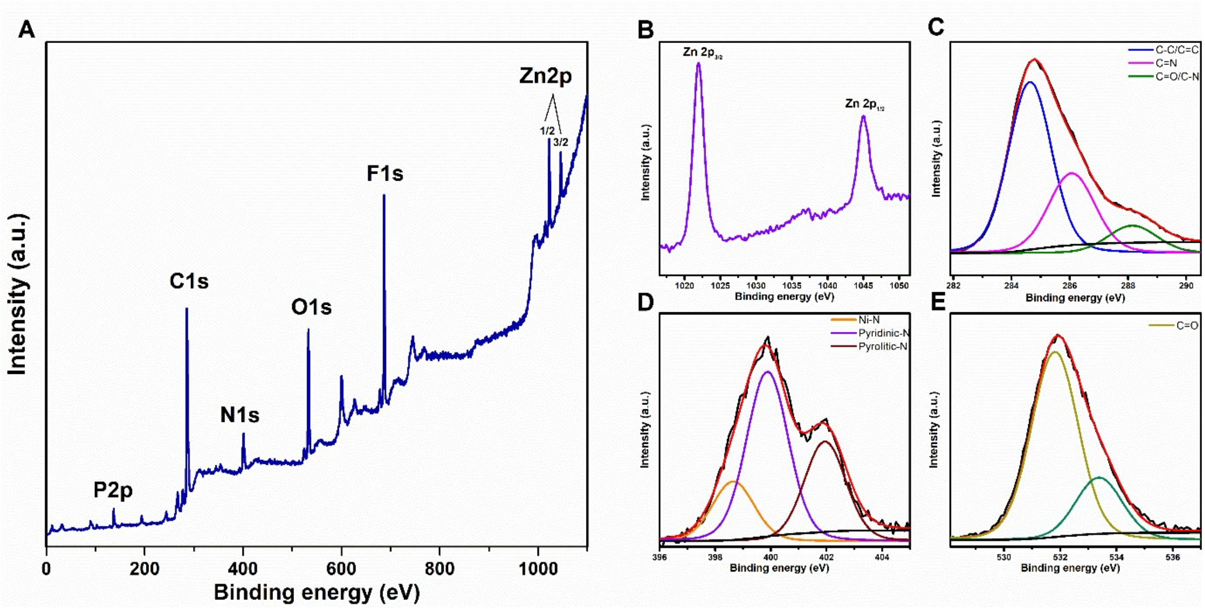

Fig S3 XPS spectra of Poly ZnEOPK (A), Ni(B), carbon(C), Nitrogen(D) and Oxygen(E).

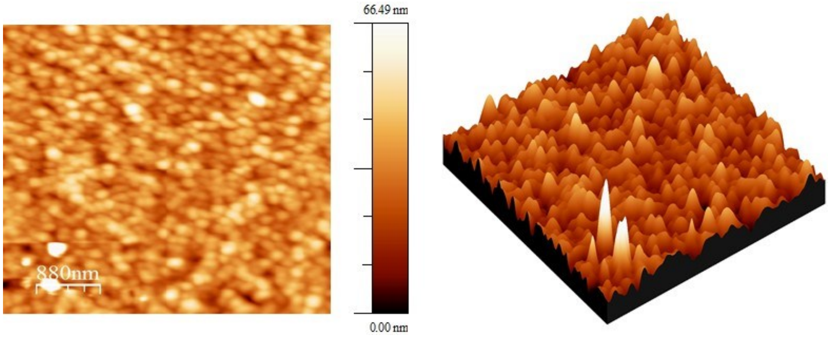

Fig S4 AFM image of poly ZnOEPK on FTO electrode as 2D (A) and 3D (B) view.

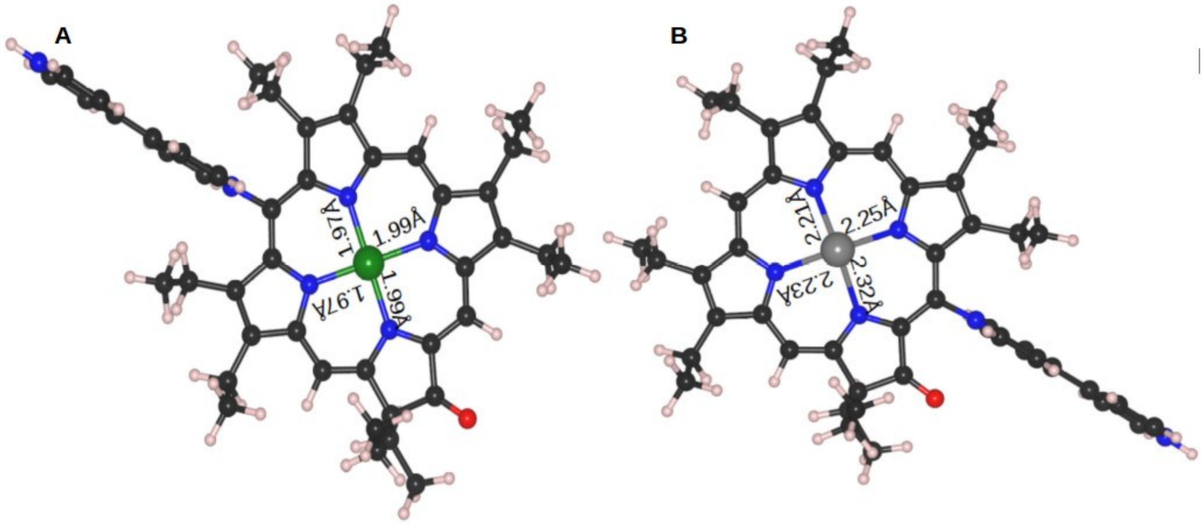

Fig S5 4,4'-Bpy attached to the meso position nearest in NiOEPK(A) farthest meso position in ZnOEPK(B) to the keto group.

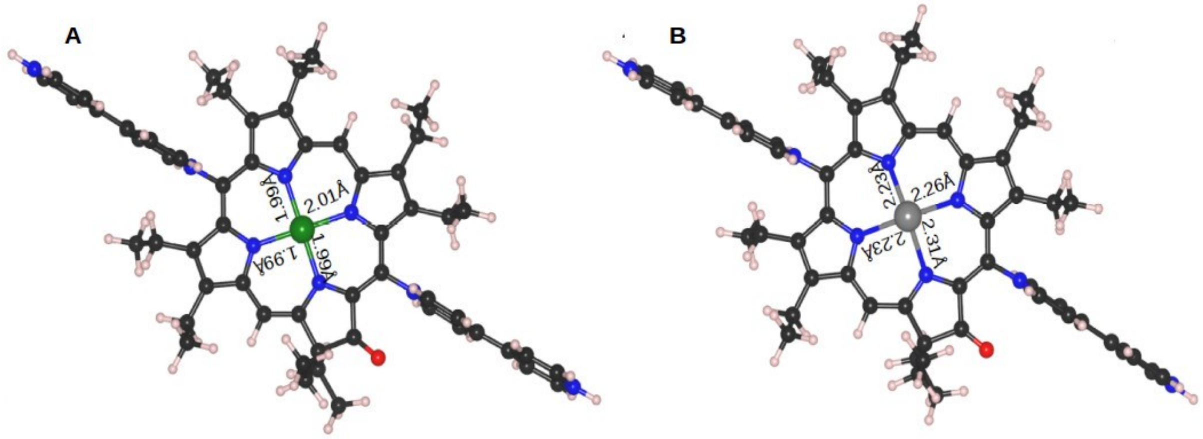

Fig S6: (**A**) second 4,4'-Bpy attached to the meso position opposite to the first one in Ni-OEPK(fig 4A) and (**B**) to the nearest meso position and opposite to the first 4,4'-Bpy unit in ZnOEPK(fig 4B).

Below tables are for the relative energies of attachment of 4, 4'-bpy on various meso positions. The energies are calculated **with reference to thermodynamically most stable position**.

Table S1: Zn 1st Bipyridine at various meso position:

**Meso position**	**dE (eV)**
20	0
5	0.0157
15	0.0248
10	0.0528

Table S2: Zn 2nd Bipyridine at various meso position :

**Meso position**	**dE (eV)**
10	0
15	0.1052
5	0.1072

Table S3: Ni 1st Bipyridine at various meso position :

Meso position	dE (eV)
10	0
15	0.0331
5	0.0493
20	0.1088

Table S4: Ni 2nd Bipyridine at various meso position:

Meso position	dE (eV)
20	0
5	0.1691
15	0.2878
